# Solar Light Elimination of Bacteria, Yeast and Organic Pollutants by Effective Photocatalysts Based on Ag/Cr-TiO_2_ and Pd/Cr-TiO_2_

**DOI:** 10.3390/nano14211730

**Published:** 2024-10-29

**Authors:** Mónica Hernández-Laverde, Nicola Morante, Blanca Liliana Gutiérrez, Julie Joseane Murcia, Katia Monzillo, Diana Sannino, Vincenzo Vaiano

**Affiliations:** 1 Grupo de Catálisis, Escuela de Ciencias Químicas, Universidad Pedagógica y Tecnológica de Colombia (UPTC), Avenida Central del Norte, Tunja 150002, Boyacá, Colombia; monica.hernandez06@uptc.edu.co (M.H.-L.); julie.murcia@uptc.edu.co (J.J.M.); 2 Escuela de Ciencias Básicas Tecnología e Ingeniería, Universidad Nacional Abierta y a Distancia (UNAD), Calle 5 #1-08, Sogamoso 152217, Boyacá, Colombia; blgutierrezp@unadvirtual.edu.co; 3 Department of Industrial Engineering, University of Salerno, Via Giovanni Paolo II 132, 84084 Fisciano, SA, Italy; nmorante@unisa.it (N.M.); kmonzillo@unisa.it (K.M.); vvaiano@unisa.it (V.V.)

**Keywords:** Cr-Ag, Cr-Pd, TiO_2_, *E. coli*, *S. cerevisiae*, sunset yellow

## Abstract

This study focused on searching for more effective nanomaterials for environmental remediation and health protection; thus, coliform bacteria, yeast and the organic food dye sunset yellow were selected as target pollutants to be eliminated under solar light by Ag/Cr-TiO_2_ and Pd/Cr-TiO_2_. Firstly, Cr^3+^ was in situ incorporated into the anatase crystalline lattice by the sol-gel method; then, Ag or Pd nanoparticles were deposited on Cr-TiO_2_ by chemical photoreduction. The scientific challenge addressed by the development of these composites was to analyse the recovery of Cr, to be employed in photocatalyst formulation and the enhancement of the TiO_2_ photocatalytic activity by addition of other noble metals. By extensive characterization, it was found that after TiO_2_ doping with chromium, the parameters of the crystal lattice slightly increased, due to the incorporation of Cr ions into the lattice. The TiO_2_ band gap decreased after Cr addition, but an increase in the optical absorptions towards the visible region after noble metals deposition was also observed, which was dependent of the Ag or Pd loading. Generally, it was observed that the noble metals type is a factor that strongly influenced the effectiveness of the photocatalysts concerning each substrate studied. Thus, by using Ag(0.1%)/Cr-TiO_2_, the complete elimination of *E. coli* from samples of water coming from a highly polluted river was achieved. Pd(0.5%)/Cr-TiO_2_ showed the highest efficiency in the elimination of *S. cerevisiae* from a lab prepared strain. On the other hand, the Pd(0.1%)/Cr-TiO_2_ sample shows the highest dye degradation rate, achieving 92% of TOC removal after 180 min.

## 1. Introduction

The increase in pollutant emission and its negative effects on the environment are important concerns worldwide [1]. For years the natural water sources have been contaminated with organic compounds such as dyes, solvents, oils, and fertilizers [2] with inorganic substances such as heavy metals, metallic compounds, inorganic salts [3], and enteric microorganisms [4,5]. These pollutants come from different anthropogenic activities. In order to reduce river pollution, a number of technologies have been studied such as heterogeneous photocatalysis, which has been demonstrated to be effective in the removal of emerging pollutants, heavy metals, organic pollutants, and pathogenic bacteria [6,7].

TiO_2_ has been the most widely studied photocatalyst due to its non-toxic nature, high stability, low cost, and abundance [8,9]. However, this semiconductor presents some disadvantages when applied at a large scale, such as: (i) the wide band gap [10], (ii) the high rate of photo-induced electron-hole pair recombination [11], and (iii) this oxide only absorbs light in the ultraviolet region of the electromagnetic spectrum, which represents approximately 5% of the entire solar spectrum [12].

There are many strategies to overcome the limitations of TiO_2_ such as: the application of metallic and non-metallic dopants [13,14,15], coupling or heterojunctions [16], and the deposition of noble metals on the titania surface such as Au [17], Ag [18], Pd [19], and Pt [20,21], which are promising pathways for the production of more efficient photocatalytic materials.

TiO_2_ doping involves the integration of transition metals into the oxide structure; this procedure replaces a certain amount of Ti atoms with doping metal ions, thus inserting new energy levels into the band structure [22]. Some researchers have suggested that the redshift is necessary to enhance optical absorption, and it could be achieved by increasing the concentration of impurity ions or oxygen defects in the TiO_2_ structure [23,24]. Thus, the main objective of doping with Cr is to decrease the extensive band gap and to modify the electronic structure of this semiconductor, which is focused on enhancing the TiO_2_ absorption of the visible region of the electromagnetic spectrum.

Additionally, the metal co-catalysis aims to decrease the electron-hole recombination and therefore enhance the photocatalytic performance of TiO_2_ [25]. In general, chemical photoreduction is employed to include plasmonic noble metals on the TiO_2_ surface, thus, the metal nanoparticles addition leads to the formation of the Schottky junction [26]. The metals can also enhance the visible excitation of surface electrons by surface plasmon resonance (SPR) effects [27]. 

It is worth mentioning that the criteria to choose Cr as a dopant for TiO_2_, lies in the fact that this metal is commonly employed in different industries as a wood protector, in the tanning of fibers, and in paintings; thus, this metal is leached in these industrial effluents. As an alternative to valorize and recycle these effluents, this metal is employed in the present study to enhance TiO_2_ light absorption.

In this study, we also selected Pd for properties such as its high activity when it interacts with the oxide’s surface [28]. The small size of highly dispersed Pd particles increases the activity concerning strong metal-support interactions [29], as well as the metal favoring the removal of organic pollutants and its antimicrobial properties, as reported in several studies [30,31]. In the case of Ag, the effect that this noble metal has on the optical and electrical properties of TiO_2_ nanoparticles [32], its high intrinsic antimicrobial activity, and the capacity of silver ions to cause the denaturation of proteins present in bacterial cell walls and to delay bacterial growth, make it an interesting choice for the design of new photocatalysts for environmental applications [33].

Based on all the aforementioned considerations, achieving a high-performance photocatalyst with an appropriate bandgap and high electron-hole pair separation efficiency is the main target of this work. Based on a previous individuation of a suitable formulation of visible light activity, this study was focused on the design of two series of photocatalysts based on Cr-doped TiO_2_. The first one was modified by Ag photodeposition and the second series of materials was obtained by Pd addition. The effect of the loading of these metals on the photocatalytic activity of the obtained materials was also evaluated by addition of Ag or Pd contents from 0.1 to 0.5 wt.%. The photocatalytic performance of the materials prepared was evaluated in the degradation of the coloring food azo dye Sunset yellow (SY FCF), and in the elimination of enteric bacteria and yeast.

## 2. Materials and Methods

### 2.1. Chemicals

Titanium tetraisopropoxide (C_12_H_28_O_4_Ti > 97% (*w*/*w*)), Sigma Aldrich, St. Louis, MO, USA), chromium (III) nitrate nonahydrate (Cr(NO_3_)_3_·9H_2_O ≥ 99%), Sunset Yellow FCF (C_16_H_10_N_2_Na_2_O_7_S_2_ 90%, Sigma Aldrich) (SY), Tetraamminepalladium (II) nitrate solution (Pd(NH_3_)_4_(NO_3_)_2_, 10 wt.% in H_2_O, 99.99%, Sigma Aldrich), Silver Nitrate (AgNO_3_, 99%, Sigma Aldrich), hydrochloric acid solution (HCl, 37% (*w*/*w*) in H_2_O, Carlo Erba, Milan, Italy), sodium hydroxide solution (NaOH, 0.5 M in H_2_O, Sigma Aldrich), and distilled water (Carlo Erba) were purchased and used as received. The molecular structure of SY is composed of benzene and naphtalene rings connected by an azo-linkage and substituted with sulphonate groups, OH, and CH_3_.

### 2.2. Materials Preparation

#### 2.2.1. Preparation of Cr-TiO_2_

The preparation method of Cr-TiO_2_ was reported in earlier works [33,34]; thus, in a general procedure, the amount of Cr(NO_3_)_3_·9H_2_O necessary to obtain a nominal Cr content of 0.7 wt.% was first dissolved in 50 mL of distilled water and this solution was mixed under magnetic stirring for 5 min. Then, 12.5 mL of C_12_H_28_O_4_Ti was dropped, under stirring at room temperature for 10 min. The suspension obtained was centrifuged and washed with distilled water three times; and finally, the powder obtained was calcined at 450 °C for 30 min, with a heating ramp of 20 °C min^−1^.

#### 2.2.2. Preparation of Pd/Cr-TiO_2_ and Ag/Cr-TiO_2_

6.25 mL of isopropanol was employed as a sacrificial agent, which was mixed with distilled water and the required amount of the metal precursor to achieve 0.1 to 0.5 wt.% of Ag or Pd with respect to the Cr-TiO_2_ content [34,35]. The resulting suspension was sonicated for 10 min in an ultrasonic bath at 20 °C. Then, the suspension was stirred for 10 min in the dark under N_2_ atmosphere. The suspension was irradiated for 2 h under continuous stirring and N_2_ flux by two UV-A lamps (240 W m^−2^). Finally, the suspension was centrifuged, and the resulting solids were dried at 90 °C for 8 h and labelled as Pd/Cr-TiO_2_ and Ag/Cr-TiO_2_ [34,35].

#### 2.2.3. Physicochemical Analysis of the Photocatalytic Materials

The photocatalytic materials prepared as previously described were characterized by different techniques and a detailed description of the experimental and equipment conditions are described below:

X-ray fluorescence (XRF) was employed to determine the chemical composition of the photocatalysts, using Panalytical Minipal PW4025 equipment (Malvern Panalytical, Westminster, London, UK). The parameters used in these analyses were: He flux, 20 KV and 180 s.

X-ray diffraction (XRD) analysis was performed with an Xpert pro Panalytical diffractometer, using the Cu Kα radiation (35 mA and 40 KV) and with a scanning angle of 2ϴ from 10 to 90° with 0.05° passes. The crystallite anatase sizes (D_Anatase_) were estimated by the Scherrer equation.

The specific surface area (S_BET_) of the prepared nanomaterials was obtained using the Costech Sorptometer 1042 analyser by the volumetric N_2_ adsorption at −196° C. Before the measurements, a degassing pretreatment of the photocatalytic particles was carried out at 150° C for 30 min in He flux.

The light absorption characteristics of the photocatalysts were studied by Diffuse reflectance UV-Vis spectrophotometry (UV-Vis DRS) in a Thermo scientific spectrometer model Evolution 300 (Thermo Fisher Scientific, Waltham, MA, USA) equipped with a diffuse reflectance BaSO_4_ covered accessory (RSA-PE-20, Labsphere Inc., North Sutton, NH, USA) and applying a reference certificated blank (SRS-99-010, Labsphere Inc., North Sutton, NH, USA). Band-gap values were calculated from Kubelka–Munk functions by the Tauc plot for indirect semiconductors [36].

Fourier Transform Infrared Spectroscopy (FTIR) analyses were carried out by using an ATR cell in a Thermo Scientific Nicolet TM iS50 FT-IR spectrometer (Thermo Fisher Scientific), samples of the photocatalysts were evaluated from 4000 to 1000 cm^−1^ with a resolution of 2 cm^−1^.

Electrochemical impedance spectroscopy (EIS) analysis of the photocatalysts was carried out using an Origalys potentiostat (ElectroChem OrigaLys, Lyon, France) and a conventional three-electrode cell equipped with an Ag/AgCl/3 M KCl reference electrode, a graphite rod was used as the counter electrode, and the working electrode. A film of the photocatalytic material was placed on the working electrode via drop-casting, wherein 20 mg of photocatalyst was mixed with 200 μL of Nafion solution (5 wt.%). The mixture coated a titanium sheet (1.5 × 1.5 cm) and was dried at 100 °C. EIS spectra were recorded from 0.1 to 100 KHz with a current amplitude of 10 mV and using a 0.5 M Na_2_SO_4_ aqueous solution as the electrolyte [37]. Measurements were performed at room temperature at open-circuit potential.

### 2.3. Photocatalytic Activity Tests

#### 2.3.1. Sunset Yellow FCF Photocatalytic Removal

The laboratory photoreactor used was a Pyrex cylindrical vessel (I.D. = 10 cm; height = 6.0 cm). A magnetic stirrer was employed to keep the photocatalyst suspended in the solution. To prevent the reaction temperature from exceeding 35 °C, a cooling fan was positioned near the reaction system. Oxygen saturation was assured by an air flow of fine bubbling.

The dye solution volume was 100 mL with an initial fixed concentration of SY ranging between 5 and 25 ppm. The spontaneous pH of the solutions containing the dye was 5.5. In some tests, the initial pH was adjusted to 4 and 11, by adding fixed amounts of HCl or NaOH concentrated aqueous solutions, respectively.

Before irradiation, the photoreactor was kept in the dark for 2 h to achieve adsorption-desorption equilibrium of SY on the catalyst surface.

**Figure 1:**
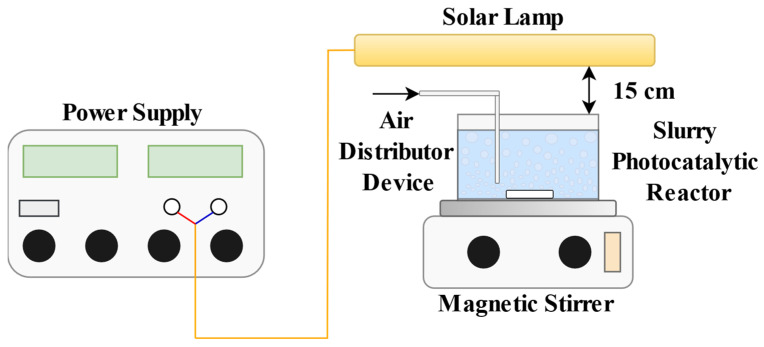
Schematic picture of the experimental setup for Sunset yellow photodegradation.

The reactor was then irradiated using two solar light lamps (Sun Glo, 8 W; wavelength range 300–900 nm; intensity 2 W m^−2^) positioned 15 cm above the reactor’s upper surface (Figure 1).

The photoreactor was covered with reflective aluminum foil to ensure only the upper surface was exposed to irradiation.

At regular intervals, approximately 3 mL of the suspension was withdrawn and centrifuged to remove catalyst particles. The aqueous solution was analysed with a UV-Vis spectrophotometer (Thermo Scientific Evolution 201) to monitor the reaction progress. Specifically, the discoloration of the dye was tracked by measuring the maximum absorbance at 485 nm [38]. The mineralization of the pollutant was assessed by measuring the total organic carbon (TOC) content in the solutions over the irradiation period. TOC was determined from CO_2_ produced by high-temperature (680 °C) catalytic combustion [39].

A kinetic analysis of the photocatalytic discoloration of SY was performed using the Langmuir–Hinshelwood model, which is typically used to describe the kinetics of photocatalytic processes [40]. The degradation rate (*r*(t)) is expressed as follows:

(1) $$r\left(t\right)=\frac{dc\left(t\right)}{dt}=\frac{k_{r}\;K_{ad}\;c}{1+K_{ad}\;c}$$

where *k_r_*, *K_ad_*, and *c*(*t*) represent the intrinsic kinetic constant, adsorption equilibrium constant, and dye concentration at a given irradiation time, respectively. Assuming weak adsorption and low compound concentrations, the above equation simplifies to a first-order kinetics expression with an apparent degradation kinetic constant (*k*):

(2) $$\ln\left(\frac{c_{0}}{c}\right)=k_{r}\;K_{ad}\;c=k\;t\;$$

The value of the apparent discoloration kinetic constant can be determined from the slope of the straight line resulting by plotting ln(*c*_0_/*c*) Vs time *t*. The TOC removal (mineralization) and SY discoloration efficiency at a given irradiation time were evaluated using the following relationship:

(3) $$TOC\;removal\;efficiency\;\left(t\right)=\left(1-\frac{TOC\left(t\right)}{TOC_{0}}\right)\;100$$

(4) $$SY\;discoloration\;efficiency\;\left(t\right)=\left(1-\frac{c\left(t\right)}{c_{0}}\right)\;100$$

where TOC(t) is the total organic carbon at a given irradiation time (mg L^−1^), *TOC*_0_ is the initial total organic carbon (mg L^−1^), and *c*_0_ is the initial SY concentration (mg L^−1^).

#### 2.3.2. Photocatalytic Treatment of Polluted River Water Samples

In these tests, water samples taken from a Colombian river called the Jordan River (geographic coordinates 5.553981, −73.350224) were studied. This river is highly polluted by industrial and domestic wastewater. The samples of the river were collected following the instructions given in the Standard Methods for the Examination of Water and Wastewater [41].

The river water samples were analysed by different physicochemical methods for the determination of chlorides, nitrates, pH, and conductivity in a Spectroquant^®^ Move 100 instrument (Merck, Darmstadt, Germany). To ensure the reproducibility of the results, each test was carried out twice.

The microbiological analysis was performed by the membrane filtration method Merck SM 9222B and by ISO 9308 method part 1 [42].

For the photocatalytic activity test on the river water, a discontinuous batch reactor was employed and the reaction parameters were: (i) 250 mL of the water sample, (ii) continuous stirring, (iii) photocatalyst dosage: 1 g L^−1^, (iv) light source: an Osram Ultra-Vitalux lamp (300 W) (Munich, Germany) with sun-like radiation spectrum in the UVA and UVB, (v) light intensity: 30 W m^−2^, (vi) oxygen flow: 0.84 STP L h^−1^, and (vii) total treatment time: 4 h.

After water treatment, the photocatalyst was recovered by filtration, and the treated water was analysed by the physicochemical and microbiological methods previously described in this section. To ensure the reproducibility of the results, all tests were carried out twice, with a standard deviation of 0.05. The arithmetic averages correspond to values reported in each test.

#### 2.3.3. Photocatalytic Elimination of Yeast

For this test, a suspension of active dry yeast (*Saccharomyces cerevisiae*) from Levapan^®^ (Bogotá, Colombia), was prepared with 3.8 × 10^5^ number yeast/mL.

The photocatalytic activity test was conducted using the prepared yeast suspension under the conditions described in the previous Section 2.3.2. After 4 h of treatment, the photocatalyst was recovered by filtration, and 1 mL of the treated sample was taken for microbiological analysis. This analysis was carried out by the plate count method by surface seeding and following the ISO 21527 method for the enumeration of yeasts and moulds—Part 1 [43]. In order to ensure the reproducibility of the results, all the analyses were performed twice.

## 3. Results and Discussion

### 3.1. Physicochemical Analysis of Photocatalytic Materials

***XRF:*** By using XRF, it was possible to confirm the effective incorporation of Cr, Ag, or Pd in the photocatalytic materials prepared.

The Cr content in the Cr-TiO_2_ material was 0.6 wt.%, which is slightly lower than the nominal content (i.e., 0.7 wt.%). On the contrary, in the case of Ag and Pd, the content of these metals in the materials obtained was slightly higher than the nominal content, thus demonstrating the effectiveness of the photodeposition method. However, the results obtained can be in the error margin of the analysis technique.

**Figure 2:**
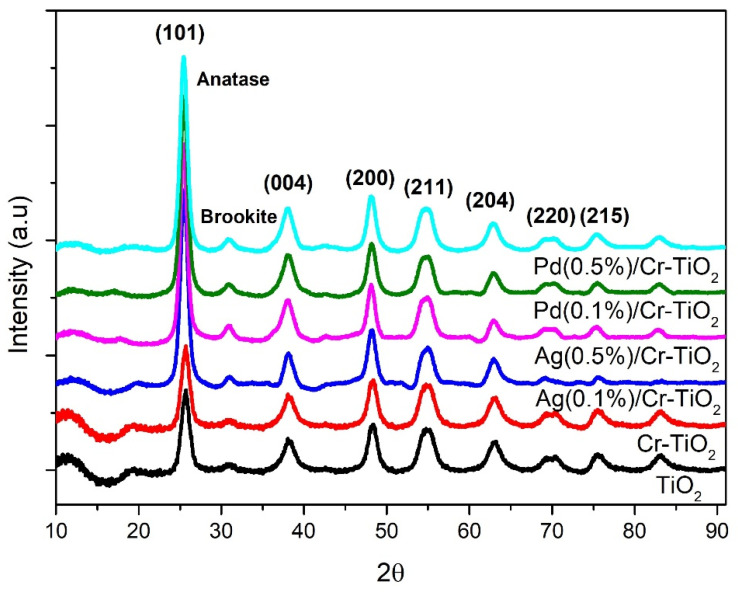
XRD patterns of all photocatalytic powders.

***XRD:*** X-ray diffraction patterns of all photocatalysts are plotted in Figure 2. As it can be seen in this figure, the samples present diffraction patterns assigned to the Anatase phase of TiO_2_ (ICDD #21-1272) with the signal of highest intensity located at 25.6° 2θ, which corresponds to the (101) plane. Peaks at 38.1°, 48.1°, 55.0°, 62.6°, 70.2°, and 75.2° correspond to the (004), (200), (211), (204), (220) and (215) planes, respectively [44]. It was also possible to observe a typical signal associated with the brookite phase located at 2θ = 31.0° [45].

On the other hand, the calculated anatase lattice parameters for planes (101) and (004) are presented in Table 1. The results obtained indicated that the doped samples present a slight increase in parameters “a”, “b”, and “c” due to the expansion of the unit cell volume compared to the bare TiO_2_, which is probably due to the lattice modification in the anatase structure arrangement by the incorporation of Cr ions into the Titania lattice. The Cr^3+^ ions can enter the lattice sites of Ti^4+^ via substitutional doping [22,46], where the expansion of the unit cell volume could be possible due to the ionic radii, which is very similar for Cr and the host titanium ions, i.e., 0.69 Å and 0.68 Å, respectively. Any significant modification of the TiO_2_ crystalline structure was observed after the addition of the metals, and similar results have been previously reported by different authors [47].

**Figure 3:**
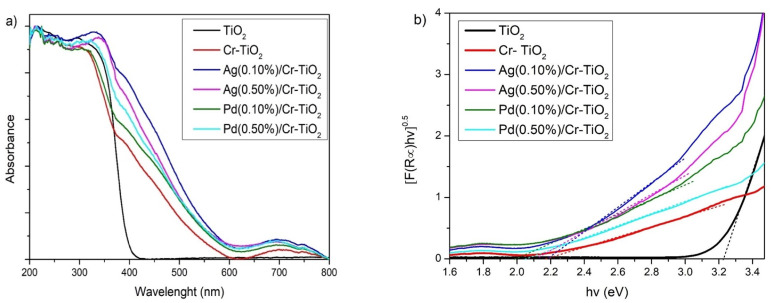
(**a**) UV-Vis DR spectra and (**b**) Band gap calculation by Kubelka–Munk functions and Tauc plots of all photocatalysts analysed.

***UV-Vis DRS***: The UV-Vis DR spectra of the synthesized photocatalyst are plotted in Figure 3. As is observed in Figure 3a, the undoped TiO_2_ exhibits absorption only at wavelengths lower than 400 nm; after its doping with Cr, the absorption in the visible region of the electromagnetic spectrum significantly increased. The red shift in the reflectance spectra of the doped TiO_2_ samples can be due to: (i) the inclusion of Cr within the network of the TiO_2_ and (ii) the change of colour of the white undoped TiO_2_ to light yellow in the Cr-TiO_2_ powders.

As observed in Figure 3a, all the Cr-TiO_2_ materials show two bands: the first one, with the highest intensity, is located between 400 and 600 nm, which is due to the ^4^A_2_ (F) to ^4^T_1_ (F) transition. The second band is located between 625 and 800 nm and can be due to the spin-forbidden ^4^A_2_ (F) to ^4^T_1_ (F) transition. In the octahedral crystalline field, Cr^+3^ ions can exhibit this type of *d-d* electronic transition [22].

On the other hand, with the incorporation of Ag or Pd nanoparticles, the visible absorption of the Cr-TiO_2_ grows even more. It is also observed in Figure 3a that the metal content has a strong influence on the width and intensity of the signals, which is mainly due to the metal nanoparticle size and distribution on the TiO_2_ surface. In general, an adsorption edge shifting toward the visible region of Titania after the deposition of Ag nanoparticles was observed, similar to that observed by other authors [48]. It was also evidenced that the silver materials exhibit higher visible absorption than that observed in the materials containing Pd, which is probably due to the more intense grey colour of the silver-based materials. Ag(0.10%)/Cr-TiO_2_ showed the highest absorption in the visible region thus indicating a potential better photocatalytic performance in this material.

The materials modified with Ag show absorption bands of charged and metallic clusters, namely Ag_n_^δ+^ and Ag_m_^0^ where n and m are low numbers, respectively; these absorptions can be observed at 220 and 365 nm in Figure 3a [49]. Ag(0.1%)/Cr-TiO_2_ and Ag(0.5%)/Cr-TiO_2_ present higher absorption at 450 nm than the Cr-TiO_2_ material, which can be due to the surface plasmon resonance (SPR) characteristic of Ag [50,51].

The addition of Pd on the surface of Cr-TiO_2_ also increased the visible light absorption which is due to the SPR of the Pd nanoparticles [52,53]. It is also evident that the Pd(0.5%)/Cr-TiO_2_ material shows higher absorption than Pd(0.1%)/Cr-TiO_2_, which was due to the higher content of Pd on the surface of the material.

Figure 3b includes the Tauc plots of the materials analysed, employed to calculate the band gap (eV) value, and the results obtained were included in Table 1. As it can be observed, the band gap value of Titania decreased after Cr doping from 3.22 to 2.15 eV, then, after the addition of Ag or Pd, the band gap of the doped material (Cr-TiO_2_) slightly decreased, thus showing the effect of the metal nanoparticles on the optical properties of TiO_2_.

The energy band structure of the catalysts is a crucial factor influencing the degradation efficiency in photocatalytic reaction systems, as it governs the redox potential of photogenerated charge carriers, which drive the catalytic reactions. In particular, the Mulliken relationship was employed to determine the edge position of the valence band (E_VB_) for TiO_2_, and Cr-TiO_2_ at pH equal to 5.5 [54,55]:

(5) $$E_{VB}=\chi -E_{c}+\frac{1}{2}E_{bg}\;$$

where χ is the electronegativity of the semiconductor and is the geometric mean of the electronegativity of the constituent atoms (the electronegativity of an atom is the arithmetic mean of the atomic electron affinity and the first ionization energy), E_c_ is the energy of free electrons on the hydrogen scale (ca. 4.5 eV), and E_bg_ is the band gap energy of TiO_2_ or Cr-TiO_2_ (eV) obtained from UV-Vis DRS analysis.

Therefore, to estimate the absolute electronegativity of the photocatalyst, we calculated the geometric mean of the electronegativities of the atoms constituting the photocatalyst:

(6) $$\chi_{TiO_{2}}={\left(\chi_{Ti}\chi_{O}^{2}\right)}^{\frac{1}{\left(1+2\right)}}$$

(7) $$\chi_{Cr-TiO_{2}}={\left(\chi_{Cr}^{0.0188}\chi_{Ti}\chi_{O}^{1.9718}\right)}^{\frac{1}{\left(0.0188+1+1.9718\right)}}$$

(8) $$\chi_{i}=\frac{1}{2}\left(E_{EA_{i}}+E_{FI_{i}}\right)$$

where $\chi_{i}$ is the absolute electronegativity of the i-th element, $E_{EA_{i}}$ is the electro-affinity of the i-th element, and $E_{FI_{i}}$ is the energy of the first ionization of the i-th element. In detail, to evaluate the absolute electronegativity of Cr-TiO_2_, the nominal Cr/Ti molar ratio for the photocatalyst (0.0188) was assumed to write the minimum formula that characterizes the doped semiconductor (TiCr_0.0188_O_1.9718_).

Finally, to determine the position of the conduction bands (E_CB_) of the synthesized samples, the following equation was used [56,57]:

(9) $$E_{CB}=E_{VB}-E_{bg}$$

**Figure 4:**
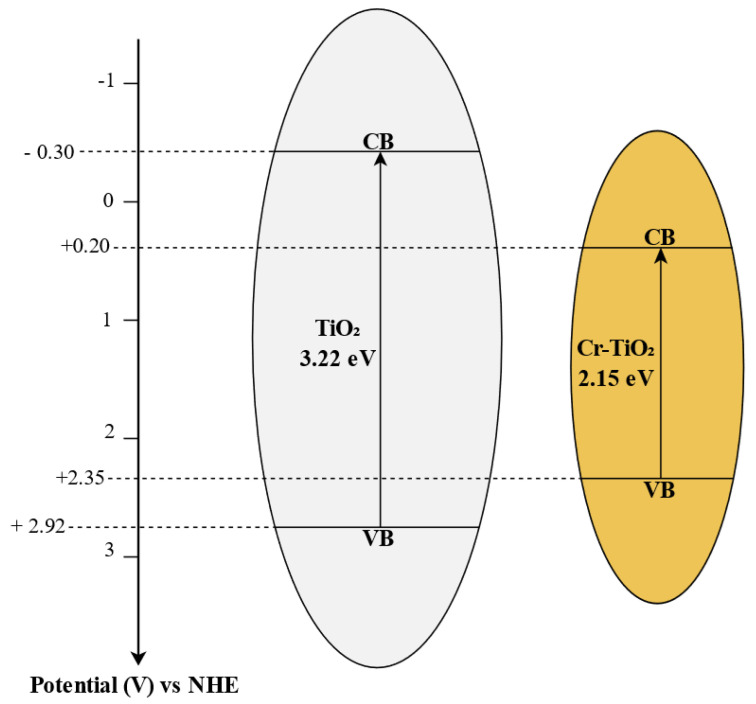
The calculated band edge positions adopted from the Mulliken electronegativity approach for (**left**) TiO_2_ and (**right**) Cr-TiO_2_ samples.

In Figure 4, the calculated band edge positions adopted from the Mulliken electronegativity approach for TiO_2_ and Cr-TiO_2_ photocatalysts are reported.

**Figure 5:**
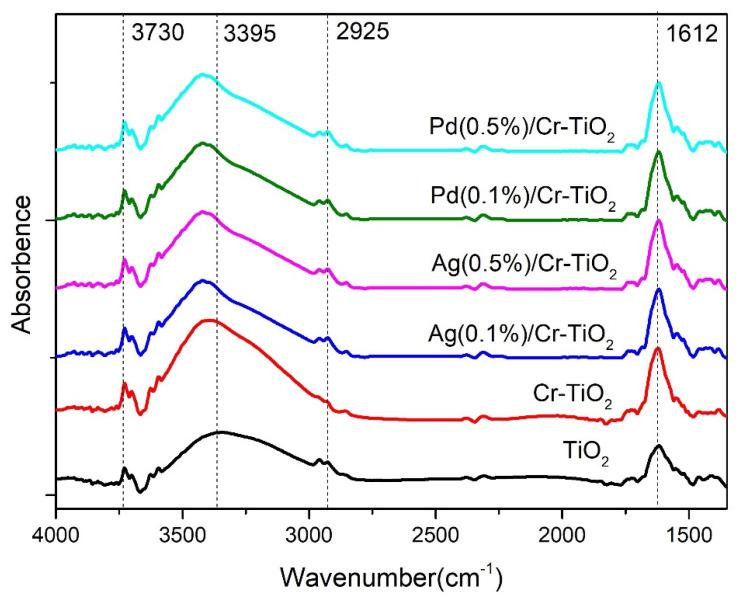
FTIR spectra of the photocatalysts analysed.

***FTIR***: Figure 5 includes the FTIR spectra of the analysed photocatalysts; in this figure is possible to identify for all the samples the presence of isolated OH^−^ groups by bands located over 3600 cm^−1^ [58].

The band centered at 3395 cm^−1^ in all the spectra is assigned to terminal Ti-OH species and the signal at 2925 cm^−1^ is assigned to adsorbed water Ti-OH_2_ [59]. The signal located at 1612 cm^−1^ in all catalysts is attributed to the water bending mode (υ2) while the broadband between 3600–3000 cm^−1^ corresponds to antisymmetric (υ3) and symmetric (υ1) stretching vibration modes of the water [60]. It is interesting to note that the intensity of the band assigned to the terminal Ti-OH species in the TiO_2_ spectrum slightly increased after Cr addition, which can indicate that this metal acts as a dopant agent in the Titania structure. Then, after the addition of Ag or Pd, the width and intensity of this band slightly decreased, which indicates that the nanoparticles of these metals are covering the Titania surface, thus leading to decreased surface hydroxylation, as has been also observed in previous works for metals such as Au or Pt [20,21].

**Figure 6:**
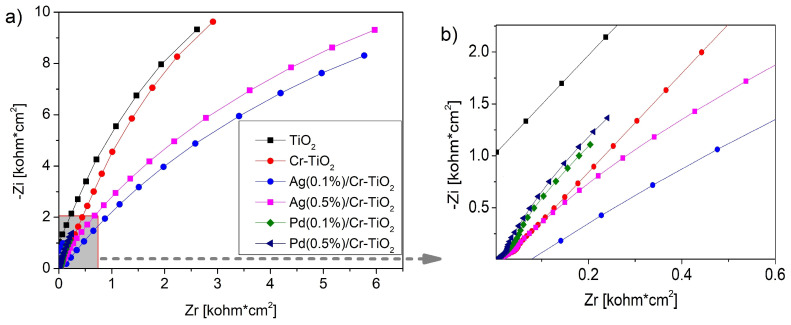
(**a**) EIS Nyquist plot of all photocatalysts and (**b**) Zoom for the EIS Nyquist plot at low frequencies.

***EIS:*** Figure 6 shows the EIS Nyquist plot of the photocatalysts analysed, this evidence the graphical representation of the real part of impedance (Zr) versus the imaginary part of impedance (Zi). Firstly, Figure 6a includes the Nyquist diagrams, and then Figure 6b shows the zoom of these diagrams at low frequencies (i.e., from 0 to 0.6). As is observed in Figure 6b, the bare TiO_2_ presented a line at high frequencies, which indicates that this material presents resistance to electronic transfer. This resistance slightly decreased after Cr doping, thus indicating that the modification of Titania with this metal improved the charge transfer.

On the other hand, the modification of Cr-TiO_2_ photocatalysts by chemical photoreduction of Pd or Ag led to even more improvement in the charge transfer. Thus, in the materials modified with Pd, a curved trend was evidenced at low frequencies; the charge transfer slightly decreased in the Pd (0.1)/Cr-TiO_2_ photocatalyst.

In the case of the materials modified by Ag addition, a curvature located at low frequencies which is greater than that observed in the Pd modified materials is evidenced in Figure 6b. This observation suggests the formation of a semicircle with a small radius, thus indicating low resistance to the charge transfer at the interface. Taking into account that the Ag(0.1%)/Cr-TiO_2_ photocatalyst presents the lowest radius arc, it is possible to assume that this material can have improved photocatalytic activity.

### 3.2. Photocatalytic Test Results

#### 3.2.1. SY Degradation Results

##### Preliminary Tests: Photolysis and Process Conducted Using TiO_2_ or Cr-TiO_2_

A preliminary test was conducted to demonstrate that SY is mineralized only due to the photodegradation activity of the photocatalysts of our reaction system. For this reason, the photolysis test was carried out in the absence of photocatalytic powders (photolysis), using a solution of distilled water with an initial SY concentration of 10 ppm, at spontaneous pH, while irradiated by the two solar lamps.

**Figure 7:**
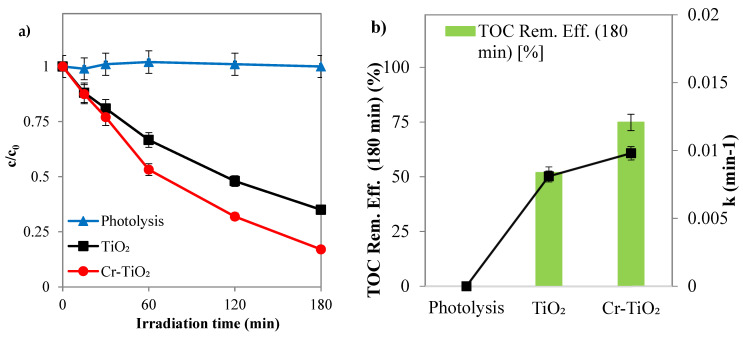
(**a**) SY discoloration and (**b**) mineralization under solar light registered for TiO_2_ and Cr-TiO_2_ compared with SY photolysis (photocatalyst dosage = 3 g L^−1^; *c*_0_ = 10 ppm; pH = 5.5).

On the other hand, the photocatalytic tests were carried out in the same operating conditions but with a photocatalyst dosage equal to 3 g L^−1^, using TiO_2_ and Cr-TiO_2_ nanopowders. The results of the tests are shown in Figure 7, from which it can be seen that photolysis alone has a negligible effect on the removal of the dye.

Cr-TiO_2_ recorded the highest values in SY discoloration and mineralization efficiencies, 87% (Figure 7a) and 75% (Figure 7b), respectively, confirming the influence of Cr metal ion doping in TiO_2_ on the intrinsic properties of the semiconductor by reducing charge carrier recombination and improving its photo-response in the solar field [61,62].

##### Influence of Pd and Ag Loading

Experimental tests were conducted to evaluate the photocatalytic activity of Pd- and Ag-based samples with varying active phase loads photodeposited on the photocatalyst; specifically, the palladium and silver loads were adjusted within the range of 0.1% to 0.5% by weight. These tests were conducted under the same operating conditions as the activity tests carried out with TiO_2_ and Cr-TiO_2_.

**Figure 8:**
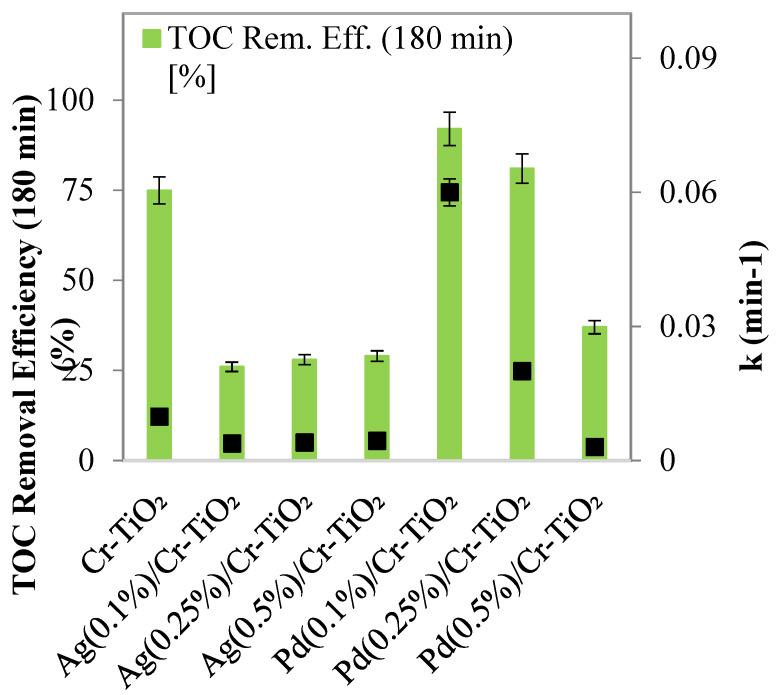
Mineralization efficiency after 180 min of solar irradiation and apparent kinetic discoloration constant values obtained using the different samples (photocatalyst dosage = 3 g L^−1^; *c*_0_ = 10 ppm; pH = 5.5).

**Figure 9:**
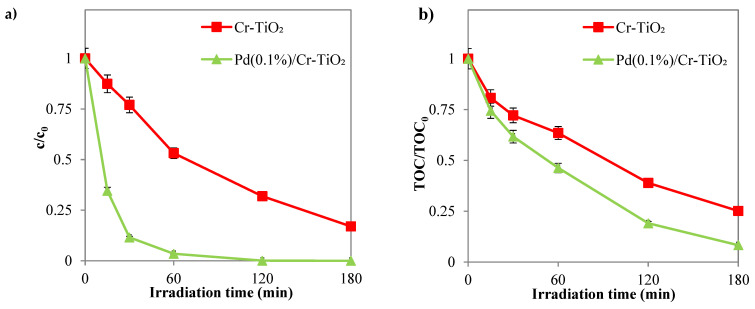
(**a**) SY discoloration and (**b**) mineralization under solar light registered with Cr-TiO_2_ and Pd(0.1%)/Cr-TiO_2_ (photocatalyst dosage = 3 g L^−1^; *c*_0_ = 10 ppm; pH = 5.5).

The results, illustrated in Figure 8, indicate that the optimal photocatalyst is Cr-TiO_2_ decorated with a palladium load of 0.1% by weight. This specific loading significantly enhances the discoloration and mineralization rates of the azo dye compared to unmodified Cr-TiO_2_ (Figure 9a,b). Indeed, it achieved the highest values for the apparent discoloration kinetic constant and TOC removal efficiency after 180 min of solar irradiation, with values of 0.06 min⁻¹ and 92%, respectively. For this reason, subsequent tests to identify the optimal operating conditions for the mineralization of SY were conducted using the Pd(0.1%)/Cr-TiO_2_ sample.

The best performance observed with this photocatalyst can be associated with its physicochemical properties, thus, as observed by UV-Vis DRS, this material presents higher absorption in the UV-visible regions of the electromagnetic spectrum than the Cr-TiO_2_ sample, which improves the radiation available for the SY photodegradation. The Pd(0.1%)/Cr-TiO_2_ sample also presents lower resistance to the charge transfer at its interface, compared with the Cr-TiO_2_ material, thus indicating improved photogenerated charges during the photocatalytic process.

On the other hand, it is important to note that the increase of the Pd content in the materials had a detrimental effect on the photocatalytic efficiency, thus, as is observed in Figure 8, the dye degradation represented by the TOC removal significantly decreases from 81 to 37% in the material containing 0.5 wt.% of Pd. In the case of the Ag/Cr-TiO_2_ material, the effectiveness in the TOC removal efficiency significantly decreased compared to pristine Cr-TiO_2_, which can be associated with the Ag particle size. These nanoparticles can be more homogeneously distributed on the Cr-TiO_2_ surface than the Pd particles, which can obstruct the light interaction with the TiO_2_ surface, and act as recombination centers.

##### Influence of Photocatalyst Dosage

**Figure 10:**
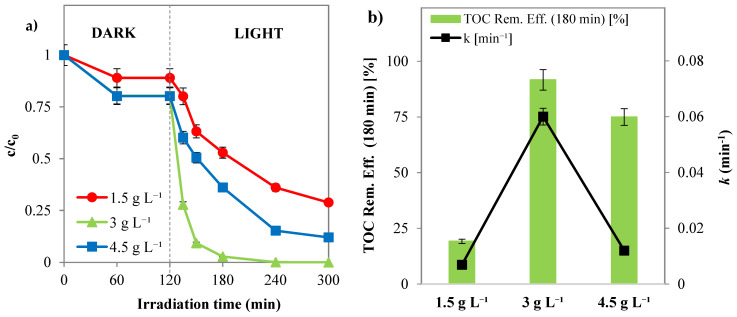
(**a**) SY discoloration and (**b**) mineralization under solar light registered with Pd(0.1%)/Cr-TiO_2_ during the experimental tests by varying the photocatalyst dosage (*c*_0_ = 10 ppm, pH = 5.5).

Since Pd(0.1%)/Cr-TiO_2_ showed the best photocatalytic performance in SY discoloration and mineralization, subsequent experimental tests were conducted with this sample. In particular, the effect of photocatalyst dosage on the SY degradation was examined and the optimal value was determined. An adequate dosage of photocatalyst can improve the photocatalytic performance and lower the energy cost of the treatment. Since the number of active sites and photo-adsorption capability of the photocatalyst have a significant impact on the photodegradation efficiency, it is a crucial parameter for photocatalytic processes [63]. From Figure 10 it can be seen that the optimal dosage of photocatalyst is equal to 3 g L^−1^, allowing the maximization of both the discoloration (Figure 10a) and the mineralization of SY, with the highest value of apparent discoloration kinetic constant of 0.06 min^−1^ (Figure 10b). Indeed, a sufficient increase in photocatalyst dosage raised the amount of photons absorbed, leading to an improvement in photodegradation rates [63,64]. Instead, when the loading was increased from 3 to 4.5 g L^−1^, photocatalytic degradation and mineralization of SY decreased. Indeed, the overdosage of the photocatalyst enhanced the opacity of the solution, decreasing the penetration of the photon flux in the reactor and, consequently, the rate of photocatalytic degradation, even in the presence of a significant number of active sites [63,65].

##### Influence of Initial Dye Concentration

**Figure 11:**
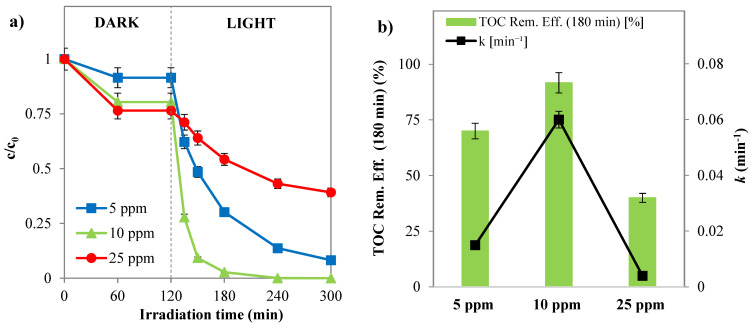
(**a**) SY discoloration and (**b**) mineralization under solar light registered with Pd(0.1%)/Cr-TiO_2_ during the experimental tests by varying the initial SY concentration (photocatalyst dosage = 3 g L^−1^, pH = 5.5).

Subsequently, the photocatalytic process at various initial SY concentrations was further evaluated using the optimal photocatalyst dosage (3 g L^−1^). From Figure 11a,b, the data indicates that 10 ppm is the optimal initial concentration for the photocatalytic reaction. Indeed, with an initial SY concentration of 10 ppm, the discoloration efficiency of 97.3% occurred after 60 min of solar irradiation, while the SY discoloration efficiency at initial concentrations of 5 and 25 ppm after 180 min of irradiation time was 91.7% and 61%, respectively (Figure 11a).

Furthermore, the results for mineralization also confirmed that 10 ppm of SY as the initial concentration is the optimal one, recording 91.6% TOC removal efficiency after 180 min of irradiation time, against 70% and 40% at 5 and 25 ppm, respectively (Figure 11b). The registered data illustrate that the pollutant discoloration and mineralization increase when the starting concentration passes from 5 to 10 ppm, but 25 ppm is recorded as the lowest photocatalytic performance. Indeed, because the adsorbed SY molecules occupy the active sites of the photocatalyst particles, an excessive increase in dye concentration inhibits the production of reactive oxygen species (ROS) such as hydroxyl radicals, superoxides, and positive holes that interact with the dye molecules. Moreover, the intensity of the incident light on the photocatalyst surface is reduced by an excessive increase in dye concentration in the solution because more photons from the solar irradiations will be absorbed by the SY molecules [34,63].

##### Influence of Initial pH

The influence of the initial pH of the aqueous SY solution on photocatalytic activity was subsequently analysed. Various tests were conducted using the Pd(0.1%)/Cr-TiO_2_ sample under previously identified optimal operating conditions. The initial pH of the solution was varied by adding a fixed amount of aqueous HCl or NaOH solution. A key factor in this analysis is the point of zero charge (PZC), which corresponds to the condition where no net charge is present on the photocatalytic surface. In fact, the PZC significantly affects the adsorption of solution species and the photocatalytic activity of materials [66]. The PZC of the optimal synthesized sample (Pd(0.1%)/Cr-TiO_2_) was determined using the mass titration method [67], registering a value of 4.8. At pH values lower than the PZC of the photocatalyst, the surface becomes positively charged due to the protonation of surface hydroxyl groups (Equation (10)). On the other hand, as the pH increases, deprotonation gradually becomes dominant (Equation (11)). When the pH exceeds the PZC, the surface of the photocatalyst becomes negatively charged [68].

(10) $$pH<PZC,Ti-OH+H^{+}⟶TiOH_{2}^{+}$$

(11) $$pH>PZC,Ti-OH+{OH}^{-}⟶TiO^{-}+H_{2}O$$

**Figure 12:**
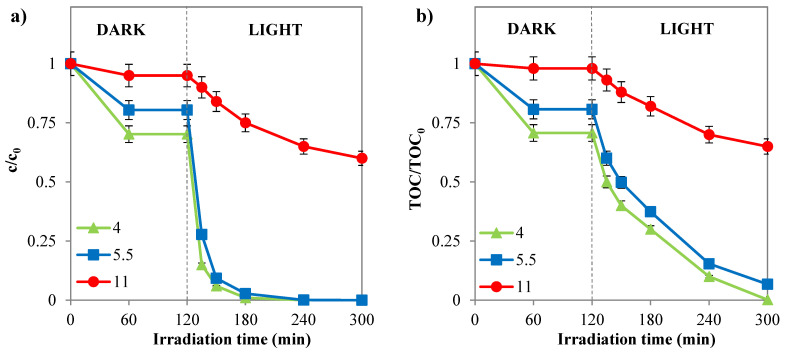
(**a**) SY discoloration and (**b**) mineralization observed with Pd(0.1%)/Cr-TiO_2_ during the experimental tests by varying the initial pH (photocatalyst dosage = 3 g L^−1^; *c*_0_ = 10 ppm).

SY is typically an anionic dye. When the pH is lower than the PZC of the photocatalyst, the sulfonate groups in SY (D–SO_3_Na) remain deprotonated, resulting in a negative charge [69]. Under acidic conditions, electrostatic attraction facilitates the adsorption of SY onto the positively charged photocatalyst surface. However, when the pH is higher than the PZC, the deprotonation of surface hydroxyl groups reduces the electrostatic attraction, leading to decreased adsorption of SY. Figure 12a illustrates that the high adsorption of the dye on the photocatalyst material causes a rapid decrease in dye concentration in the acidic pH range. These results indicate that the initial pH of the solution influences the kinetics of the photocatalytic process by affecting the amount of dye adsorbed on the photocatalyst. The optimal initial pH for achieving the highest dye discoloration and mineralization is 4 (Figure 12a,b). Under these conditions, the reaction system achieved complete discoloration after 60 min and mineralization of the azo dye within 180 min of solar irradiation.

##### Electric Energy Consumption Evaluation

Finally, the electric energy consumption associated with the photodegradation of 90% of SY in 1 m^3^ of water contaminated with the azo dye was assessed under optimal operating conditions. This analysis utilized the correlation proposed by Bolton et al. [70], expressed as:

(12) $$E_{E/O}=\frac{P\;t_{90\%}\;1000}{V\;60\ln\left(\frac{c\left(t_{0}\right)}{c\left(t\right)}\right)}$$

where *E_E/O_* is the electric energy consumption (kWh), *P* is the nominal power of the light source (kW), *t*_90%_ is the irradiation time to achieve 90% removal of SY (min), *V* is the volume of treated solution (L), *c*(*t*_0_) is the concentration of SY (ppm) at the start of irradiation, and *c*(*t*) is the concentration of SY (ppm) at generic time *t*.

Next, the following best-performing literature works on the degradation of SY have been identified:

- W1 [71]: The photocatalytic reaction was conducted in a reactor under visible light irradiation using a 200 W tungsten bulb from Osram. Optimal performance was achieved with the addition of 800 mg of CuCr_2_O_4_ photocatalytic nanoparticles to 0.05 L of SY aqueous solution.
- W2 [72]: The photocatalytic performance of a Pd-BiFeO_3_ composite was evaluated for the degradation of SY under visible light irradiation. The experiments were conducted in a photoreactor illuminated by a 105 W visible lamp. Each test analysed the photodegradation of 100 mL of dye aqueous solution, starting with an initial SY concentration of 10 ppm.
- W3 [73]: The photocatalytic activity of the TiO_2_/CAC sample was evaluated in a reactor under UV-A light using four parallel medium-pressure mercury lamps (8 W). Specifically, 50 mL of SY solution with an appropriate amount of catalyst was tested in each experiment.
- W4 [72]: A slurry reactor containing a suspension of HoAG_5_/g-C_3_N_4_ nanocomposite was used, irradiated by a 350 W xenon-mercury lamp. The volume of the SY aqueous solution was 100 mL.
- W5 [74]: The degradation experiment with the best performance was conducted by treating 200 mL of SY aqueous solution with an initial concentration of 10 ppm. The BiOBr dosage was 1.31 g L^−1^, and the system was irradiated using a 55 W light bulb as the visible light source.
- W6 [75]: The mineralization of SY using a ZnS-TiO_2_ photocatalyst was evaluated under UV-A light, provided by four parallel medium-pressure mercury lamps (8 W each). Optimal performance was observed with a 50 mL SY solution (~450 ppm) when the photocatalyst dosage was set to 5 g L^−1^.
- W7 [76]: The photocatalytic activity of selenium nanoparticles (Se-NPLs) was evaluated in a slurry reactor under UV-C light, utilizing an 8 W Herolab lamp. Each batch experiment involved using a 100 mL beaker containing 40 mg of Se-NPLs and 100 mL of a 5 mg L^−1^ aqueous SY solution.

From the data of the seven works in the literature, the apparent discoloration kinetic constant of the dye was identified. Additionally, the electricity consumption required for a 90% reduction of the pollutant in 1 m^3^ of solution was calculated using Equation (12) and compared to the consumption required by the reaction system of the present work under the following conditions: photocatalyst dosage = 3 g L^−1^, *c*_0_ = 10 ppm, and initial pH = 4. The results are presented in Table 2.

The experimental results demonstrated that using small quantities of palladium significantly enhances the photodegradation process of azo dyes under solar irradiation. Our photocatalytic system exhibits the highest energy efficiency in SY removal, with the lowest energy consumption for 90% pollutant removal at 38.1 kWh m^−3^.

##### Analysis of the Possible Reaction Mechanism

The photocatalytic activity tests were subsequently conducted to investigate the reaction mechanism for the mineralization of the azo dye, using a fixed initial concentration of specific scavenger probe molecules. These experiments were performed under optimal conditions determined from previous tests, with an initial SY concentration of 10 ppm and a Pd(0.1%)/Cr-TiO_2_ dosage of 3 g L⁻¹.

The scavenger probe molecules employed included isopropanol (IPA, 10 mM) to avoid the presence of hydroxyl radicals (OH^•^) [77,78,79], benzoquinone (BQ, 1 µM) for superoxide radicals (O_2_^−•^) [80,81,82] and methanol (MetOH, 10 mM) for positive holes (h^+^) [83,84].

**Figure 13:**
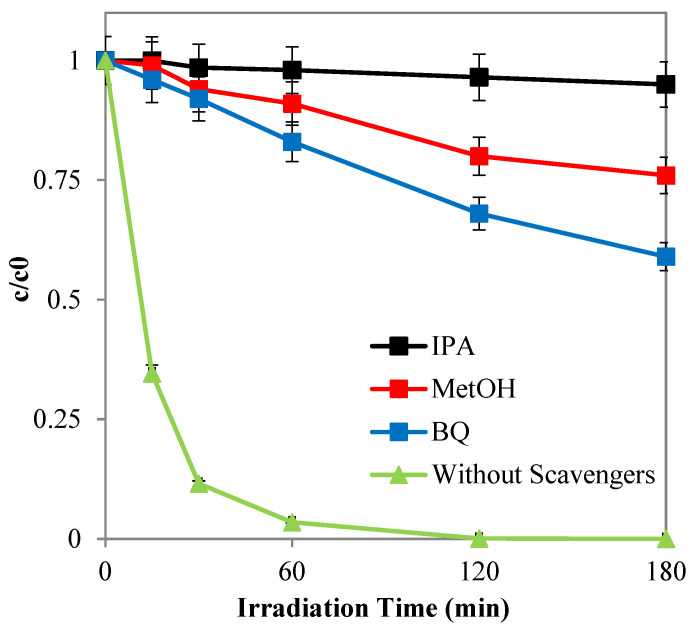
SY discoloration observed with Pd(0.1%)/Cr-TiO_2_ during the experimental tests with the presence of IPA, MetOH, and BQ, and without the scavengers.

The results shown in Figure 13 depict a significant reduction in photocatalytic activity when the formation of hydroxyl and superoxide radicals is inhibited. Specifically, in the presence of isopropanol, which scavenges hydroxyl radicals, the SY apparent degradation kinetic constant decreased by 95% compared to the control test conducted under identical conditions without the scavenger. These experimental data underline that the degradation of the azo dye using the Pd(0.1%)/Cr-TiO_2_ primarily occurs through hydroxyl radicals which are generated by both positive holes and superoxide ions. However, the Pd(0) photodeposited on the surface of the photocatalytic nanoparticles acts as an electron collector allowing the formation of H_2_O_2_, increasing the hydroxyl radicals for the oxidation of pollutant [85,86].

The following reactions describe a potential mechanism for the mineralization of SY:

(13) $$Pd\left(0.1\%\right)/Cr-{TiO}_{2}+h\nu \to h^{+}+e^{-}$$

(14) $$O_{2}+e^{-}\to O_{2}^{-•}$$

(15) $$OH^{-}+h^{+}\to OH^{•}$$

(16) $$O_{2}^{-•}+H^{+}\to HO_{2}^{•}$$

(17) $$HO_{2}^{•}+HO_{2}^{•}\to H_{2}O_{2}+O_{2}$$

(18) $$H_{2}O_{2}+e^{-}\to OH^{•}+OH^{-}$$

(19) $$SY+OH^{•}\to intermediates\to CO_{2}+H_{2}O$$

(20) $$e^{-}+h^{+}\to E+N$$

where N is a neutral center, and E is the energy generated by the recombination of the photoexcited pairs (light hν′≤hν, or heat).

#### 3.2.2. Photocatalytic Treatment of Polluted River Water

The effectiveness of the photocatalysts prepared with 0.1 and 0.5 wt.% of Ag or Pd were also evaluated in the treatment of water samples from a polluted river. Different physicochemical parameters were evaluated before and after photocatalytic treatment on the water river samples and the main results are presented in Table 3.

As observed in Table 3, the pH value slightly increased after the photocatalytic treatments, which is due to a mild alkalinity of the reaction medium by the hydroxyl groups generated during the photocatalytic process [87]. Other physicochemical parameters such as chlorides, nitrates, and conductivity also decrease after treatments, which is mainly due to the adsorption of ionic species such as NO^3−^, Cl^−^, and CO_3_^2−^ on the surface of the photocatalytic materials, thus reducing the concentration of pollutants in the fluid phase [88]. The Pd(0.5%)/Cr-TiO_2_ exhibited the best performance in surface ion adsorption, which can be related to its highest surface area compared with the materials modified with metals.

**Figure 14:**
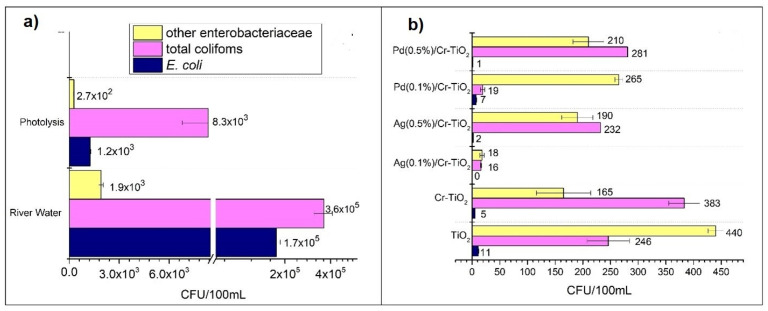
Population of bacteria in the water river sample, (**a**) before and after photolysis, (**b**) after the different photocatalytic treatments.

The content of enteric bacteria (i.e., total coliforms, *E. coli*, and other Enterobacteriaceae) in the river water sample was also analysed before and after the photocatalytic treatment, and the results obtained are represented in Figure 14. As observed in Figure 14a, and as expected, the UV-Vis light presents a certain bactericidal effect, thus leading to a decrease in the bacteria content in the photolysis test which was carried out in the presence of radiation and without photocatalytic powders in the reaction medium. Then, as observed in Figure 14b, the bacteria removal significantly increased by close to 99% in the tests performed in the presence of the photocatalytic materials.

It is also observed in Figure 14a that the efficiency of TiO_2_ in the *E. coli* and other Enterobacteria removal slightly improved by Cr doping, thus showing the improved optical properties of this material (increased light absorption in the visible region and decreased band gap) as it was determined by UV-Vis DRS analysis previously described.

When Ag or Pd was added to Cr-TiO_2_, the bacteria removal efficiency was improved, which is mainly due to the effect of the metallic particles acting as electron traps, thus decreasing the photogenerated charges recombination.

It is interesting to note that the substrate to be treated is an important factor influencing the photocatalytic performance in the materials evaluated; thus, different from the results observed in the SY degradation (Section 3.2.1). In the water river treatment, the best performance was observed with the silver modified materials. Thus, the total elimination of *E. coli* was only achieved by using Ag(0.1%)/Cr-TiO_2_ in the photocatalytic treatment. With this material, the highest elimination of total coliforms and other Enterobacteriaceae was also obtained. The results obtained can be explained by taking into account the well-known bactericidal effect of the silver nanoparticles compared with the Pd; likewise, as observed by EIS analysis, the Ag(0.1%)/Cr-TiO_2_ presented the lowest charge transfer resistance, thus demonstrating the highest effectiveness of this material in the river water sample treatment. It was also observed that an increase in the loading of Ag or Pd from 0.1% to 0.5% had a detrimental effect on the biocidal efficiency of the Cr-TiO_2_ photocatalyst, which can be due to the obstruction of active sites on the surface, which could affect the amount of reactive oxygen species (ROS) in the reaction medium.

On the other hand, in order to ensure the real effectiveness in the *E. coli* inactivation, regrowth tests were also performed by storing the treated water at room temperature for 24 h. After this time passed, the bacteria content was analysed, and no *E. coli* regrowth was observed, thus indicating the effectiveness of the treatment over time. However, it is important to consider that total coliforms and other enterobacteria still remained after treatment.

#### 3.2.3. Photocatalytic Test of Yeast Elimination

**Figure 15:**
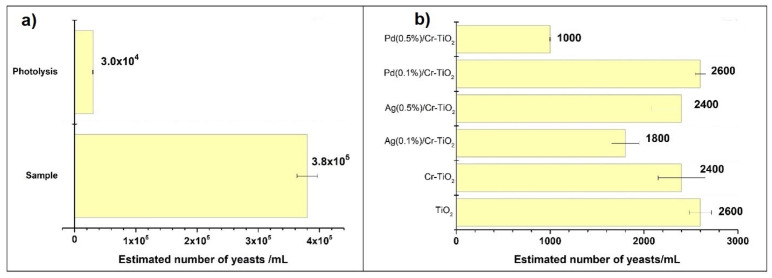
Yeast content (**a**) Sample and after photolysis, (**b**) after all photocatalytic treatments.

It is also interesting to consider that different microorganisms can be more or less sensitive to the photocatalytic treatment. For that reason, we decided to compare two completely different microorganism species, the first one the enteric bacteria previously reported, and in this section the yeast photocatalytic inactivation. *S. cerevisiae* presents different properties in the cell wall compared to gram-negative bacteria studied in Section 3.2.2 and the results obtained in its elimination are presented in Figure 15.

As observed in this figure and similar to that observed in the bacteria elimination, the UV-Vis light also has a negative effect on the yeast cell, thus leading to a decrease in the cell content after the photolysis test (Figure 14a). On the other hand, the yeast elimination significantly increased after photocatalytic treatment (Figure 14b), and the effectiveness tendency in the analysed materials exhibits the following order: Pd(0.5%)/Cr-TiO_2_ > Ag (0.1%)/Cr-TiO_2_ > Cr-TiO_2_ > Ag (0.5%)/Cr-TiO_2_ > Pd (0.1%)/Cr-TiO_2_ > TiO_2_.

In general, it was observed that the yeast presented more resistance to be eliminated by photocatalysis compared with *E. coli*, which can be due to the structural differences presented in the cell walls of these microorganisms. Thus, the yeast cell wall is thicker compared with bacteria; likewise, yeast is composed of dry cell mass (15–25%) [89], β-glucan (29% to 64%), mannan (31%), protein (13%), lipids (9%), and chitin (1–2%) [90,91]. Indeed, this highlights the significant protection provided by the yeast cell wall to the internal organelles of the cell during the photocatalytic treatment.

The damage caused to the yeast cell wall by ROS leads to cell inactivation, as evidenced by the decrease in the estimated number of yeast cells/mL after photocatalytic treatment. However, there is a possibility that the by-products of cell wall rupture, such as β-glucan and mannan, create a chemical environment unfavorable for the generation of ROS. Some studies have shown that the hydroxyl groups of β-glucan and mannan combine to inhibit the generation of hydroxyl radicals [92,93].

This could explain why the photocatalytic materials were less effective in yeast elimination. It was also important to highlight that yeast can generate mechanisms of resistance to oxidative stress, which prevents complete elimination from occurring.

In this case, it was important to highlight the biocidal effect of Pd NPs, as the most effective material was Pd(0.5%)/Cr-TiO_2_. The mechanism of action can be explained from two perspectives: (i) the small size of Pd NPs allows them to contact the yeast cells, and the metal ions tend to penetrate inside the cell, causing oxidative stress that leads to cell death [94], and (ii) previously, authors have reported the ability of Pd to generate reactive oxygen species (ROS) [95]. This could promote the rupture of the yeast cell wall by ROS generated from the metal and ROS from TiO_2_.

### 3.3. Discussion on Photocatalytic Results

The results in the elimination of the different pollutants point out that a high efficiency with the application of solar light could be obtained. In the case of organic anionic food dye SY, the boost to its decolorization and mineralization is due to the selection of Pd as a co-catalyst with respect to silver on Cr-TiO_2_. To explain the higher activity of Pd, it could be considered that a higher work function of this metal increases the height of the Schottky barrier with respect to silver, leading to a more favorable electron-hole separation. As demonstrated by the scavenger tests, the most active ROS species is the OH radical, generously generated by the interaction of the photogenerated holes with OH- adsorbed groups, and interaction with the adsorbed water leads to almost total mineralization of SY FCF. However, an increase in the Pd content induces a slight increase in the band gap values, resulting in a reduction of photoactivity. The optimal load of Pd is concluded to be 0.1 wt.%. Concerning the microorganism, Ag performs better than Pd owing to its capacity to also generate ROS in the dark and to its high toxicity to the bacteria, again with an optimal low load of 0.1 wt.% on Cr-TiO_2_, although Pd(0.1%)/Cr-TiO_2_ had comparable results in the removal of *E. coli* and total coliform and an Enterobacteriaceae conversion higher than 90% in the water river sample. With regards to yeast removal, the increase of Pd presence on Cr-TiO_2_ induces the highest value of 96.67%, but all the photocatalysts show high ability in their elimination, with performances higher than 90%.

On the basis of these considerations, a suitable balance among the limiting of the costs of the photocatalyst and the high photoactivities showed allows Pd(01%)/Cr-TiO_2_ to be considered as the optimal composition for a strong reduction of the four polluting agents under solar light.

## 4. Conclusions

The Cr-TiO_2_ photocatalysts were successfully synthesized by the sol-gel method, and some physicochemical properties of this material were improved by chemical photoreduction of Ag or Pd nanoparticles. Thus, the heterojuncted nanomaterials presented higher absorption in the visible region of the electromagnetic spectrum and higher charge transference compared with the bare TiO_2_.

The functionalizing photocatalysts with noble metals are highly effective for maximizing the efficiency of photocatalytic degradation of SY FCF azo dye and other micropollutants. Among the different compositions, Pd(0.1%)/Cr-TiO_2,_ with the lowest E_BG,_ emerged as a powerful nanomaterial able to give a very high and desired mineralization of the organic dye.

Indeed, in the study of photocatalytic activity for bacteria removal, it was determined that Ag(0.1%)/Cr-TiO_2_ exhibited the highest efficiency. Thus, the minimum amount of Ag nanoparticles is sufficient to achieve complete elimination of *E. coli.*

Regarding the elimination of *S. cerevisiae*, the material that showed the best performance was Pd(0.5%)/Cr-TiO_2_. In this case, the Pd nanoparticles play a crucial role in their affinity with the cell and the biocidal effect of the metal. The higher amount of 0.5% showed better results compared to the same material in lower amounts. Similarly, the larger surface area of this material could enhance the surface adsorption of ions such as nitrate, chloride, and carbonate present in river water.

Despite these differences, Pd-based nanomaterial demonstrated activities higher than 90% with respect to the removal of all tested pollutants. The lower amount of Pd is sufficient to guarantee an efficient and low-cost photocatalyst for future solar applications.

This study demonstrated that the formulation of a solar photocatalyst for the removal of different natural pollutants can be finely tuned to get more advantageous results when they prevail within wastewater.

## Figures and Tables

**Table 1:** Main photocatalysts characterization results.

Photocatalysts	D_Anatase_ (nm)	S_BET_ (m^2^g^−1^)	Lattice Parameters (A°)		Band Gap (eV)
			a = b	c	
TiO_2_	7.71	107	3.69	9.33	3.22
Cr-TiO_2_	7.59	113	3.73	9.41	2.15
Ag (0.1%)/Cr-TiO_2_	7.68	91	3.75	9.44	2.16
Ag (0.5%)/Cr-TiO_2_	8.14	92	3.75	9.44	2.12
Pd (0.1%)/Cr-TiO_2_	7.66	95	3.75	9.44	2.00
Pd (0.5%)/Cr-TiO_2_	7.74	103	3.75	9.44	2.03

**Table 2:** Comparison of the energy cost for the degradation of 90% of the Sunset Yellow FCF in 1 m^3^ of solution for our reaction system at the optimal operative conditions and the other works reported in the literature.

Photocatalyst	Type of Light	*P* (kW)	*k* (min^−1^)	*t*_90%_ (min)	*V* (L)	*E_E_*_/*O*_ (kWh m^−3^)	System
CuCr_2_O_4_	Visible Light	0.20	0.014	164.5	0.05	4761.9	[70]
Pd-BiFeO_3_	Visible Light	0.11	0.017	136.2	0.1	1035.5	[71]
TiO_2_/CAC	UV-A	0.03	0.039	59.0	0.05	273.5	[75]
HoAG5/g-C_3_N_4_	Visible Light	0.35	0.037	61.6	0.1	1559.7	[73]
BiOBr	Visible Light	0.06	0.007	343.7	0.2	684.1	[74]
ZnS-TiO_2_	UV-A	0.03	0.040	57.6	0.05	266.7	[75]
Se-NPLs	UV-C	0.01	0.002	1151.3	0.1	666.7	[76]
Pd(0.1%)/ Cr-TiO_2_	Solar Light	0.02	0.070	32.9	0.1	38.1	Our system

**Table 3:** Water river Quality Control Parameters analysed before and after photocatalytic treatment.

Treatment	pH	Chlorides (mg/L)	Nitrates (mg/L)	Conductivity (µS/cm)
Starting river water sample	6.78	118	1.3	119.2
Photolysis	6.60	116	1.2	118.8
TiO_2_	7.03	27	0.6	24.1
Cr-TiO_2_	6.91	24	1.1	79.3
Ag (0.1%)/Cr-TiO_2_	6.61	16	1.1	87.0
Ag (0.5%)/Cr-TiO_2_	6.85	20	0.9	82.1
Pd (0.1%)/Cr-TiO_2_	6.92	22	1.1	16.2
Pd (0.5%)/Cr-TiO_2_	6.79	<3	0.3	12.5

## Data Availability

Data are contained within the article.
